# An Assay Combining Droplet Digital PCR With Propidium Monoazide Treatment for the Accurate Detection of Live Cells of *Vibrio vulnificus* in Plasma Samples

**DOI:** 10.3389/fmicb.2022.927285

**Published:** 2022-07-15

**Authors:** Ling Hu, Yidong Fu, Shun Zhang, Zhilei Pan, Jiang Xia, Peng Zhu, Jing Guo

**Affiliations:** ^1^Hangzhou Medical College, Hangzhou, China; ^2^Ningbo Stomatology Hospital, Ningbo, China; ^3^Hwa Mei Hospital, University of Chinese Academy of Sciences, Ningbo, China; ^4^Ningbo Institute of Life and Health Industry, University of Chinese Academy of Sciences, Ningbo, China; ^5^Key Laboratory of Diagnosis and Treatment of Digestive System Tumors of Zhejiang Province, Ningbo, China; ^6^Pilot Gene Technologies (Hangzhou) Co., Ltd., Hangzhou, China

**Keywords:** *Vibrio vulnificus*, droplet digital PCR, propidium monoazide, *vvhA* gene, clinical, accurate detection

## Abstract

*Vibrio vulnificus* (*V. vulnificus*) is one of the most common pathogenic *Vibrio* species to humans; therefore, the establishment of timely and credible detection methods has become an urgent requirement for *V. vulnificus* illness surveillance. In this study, an assay combining droplet digital PCR (ddPCR) with propidium monoazide (PMA) treatment was developed for detecting *V. vulnificus.* The primers/probes targeting the *V. vulnificus* hemolysin A (*vvhA*) gene, amplification procedures, and PMA processing conditions involved in the assay were optimized. Then, we analyzed the specificity, sensitivity, and ability to detect live cell DNA while testing the performance of PMA-ddPCR in clinical samples. The optimal concentrations of primers and probes were 1.0 and 0.3 μM, respectively. The annealing temperature achieving the highest accuracy in ddPCR assay was 60°C. With an initial *V. vulnificus* cell concentration of 10^8^ CFU/mL (colony-forming units per milliliter), the optimal strategy to distinguish live cells from dead cells was to treat samples with 100 μM PMA for 15 min in the dark and expose them to LED light with an output wavelength of 465 nm for 10 min. The specificity of the PMA-ddPCR assay was tested on 27 strains, including seven *V. vulnificus* strains and 20 other bacterial strains. Only the seven *V. vulnificus* strains were observed with positive signals in specificity analysis. Comparative experiments on the detection ability of PMA-ddPCR and PMA-qPCR in pure cultures and plasma samples were performed. The limit of detection (LOD) and the limit of quantitation (LOQ) in pure culture solutions of *V. vulnificus* were 29.33 and 53.64 CFU/mL in PMA-ddPCR, respectively. For artificially clinical sample tests in PMA-ddPCR, *V. vulnificus* could be detected at concentrations as low as 65.20 CFU/mL. The sensitivity of the PMA-ddPCR assay was 15- to 40-fold more sensitive than the PMA-qPCR in this study. The PMA-ddPCR assay we developed provides a new insight to accurately detect live cells of *V. vulnificus* in clinical samples, which is of great significance to enhance public health safety and security capability and improve the emergency response level for *V. vulnificus* infection.

## Introduction

*Vibrio vulnificus* (*V. vulnificus*), one of the notorious pathogenic *Vibrio* species in Vibrionaceae, can be found in seawater and aquatic products near the coast in tropical and subtropics (Guan et al., 2021). It could enter the human body through the consumption of raw, under-cooked seafood (like oysters, fish, and shellfish), contact with the open wound on skin, or the bite of insects (Liang et al., 2021). *V. vulnificus* could cause a wide range of human diseases, such as septicemia (Narendrakumar et al., 2021), wound infections (D’Souza et al., 2018), necrotizing fasciitis (Tsai et al., 2022), meningoencephalitis (He et al., 2019), and acute gastrointestinal reactions (Hannet et al., 2019). Wound infections (mean incubation period of 16 h) and septicemia (mean incubation period of 26 h) caused by *V. vulnificus* deteriorate very quickly and could be easily misdiagnosed (Baker-Austin and Oliver, 2020), which results in high mortality rates for *V. vulnificus* infection over 24 h (Yun and Kim, 2018). The accurate diagnosis and debridement of *V. vulnificus* infections are crucial to significantly reduce the mortality and amputation rate of patients with serious infections, such as necrotizing fasciitis (Yu et al., 2017). There are growing pieces of evidence (Lee et al., 2019; Brehm et al., 2021; Kim and Chun, 2021) that *V. vulnificus* is posing a more serious threat to human health due to the increasing seawater temperatures in many areas of the world. Additionally, the increase in seafood farming and global seafood trade has led to an increase in exposure risk of *V. vulnificus* (Liang et al., 2021). Hence, the establishment of rapid and credible detection methods for *V. vulnificus* has become more important in *Vibrio* illness surveillance.

The conventional clinical detection of *V. vulnificus* is based on the microbiological culture method. The complex processes, including incubation, selective plate isolation, and biochemical identification, usually require several days, which not only is time-consuming (Bonny et al., 2022) but also might cause interference to the identification of *V. vulnificus* due to over-proliferation of the other bacteria in the samples from blood and infected trauma tissue culture fluid. In addition, when *V. vulnificus* is exposed to adverse growth conditions, such as lower temperature (below 13°C), nutrient deficiency, and disinfectants, it often exists in a viable but non-culturable (VBNC) state (Malayil et al., 2021). However, the traditional pathogenic culture method cannot detect VBNC cells (Zhao et al., 2017), which might result in false-negative results and increase the risk of transmission of *V. vulnificus* infection. Antibiotics may also affect the results of the pathogenic culture. Some researchers in a clinical study (Lee et al., 2017) analyzed the blood culture results from 23 hospitalized cases of *V. vulnificus* infection and found that the culture method could not detect *V. vulnificus* in the blood samples of eight patients who used antibiotics before sample collection. The immunoassay method (Rengpipat et al., 2008; Jadeja et al., 2010) for the detection of *V. vulnificus* is also questionable due to the difficulty of obtaining antibodies, easy inactivation during storage, and the *Vibrio*-associated cross-antigens (Lee et al., 2021b), and the high cost of antibody production also leads to difficulties in practical application (Harwood et al., 2004). The traditional pathogenic culture method and immunoassay method are cumbersome, unreliable, and prone to false-negative results, especially in distinguishing homologous strains and VBNC cells. It is necessary to find more accurate detection techniques for *V. vulnificus* detection (Copin et al., 2021). In recent years, with the development of heat-resistant DNA polymerases (e.g., *Taq* DNA polymerase) and thermal cycling instruments, molecular biology techniques represented by polymerase chain reaction (PCR) have gradually advanced from the research laboratories. Currently, the emergence of quantitative real-time PCR (qPCR) methods has greatly promoted the application of PCR in the quantitative detection of clinical pathogenic microorganisms (Cao et al., 2019; Guan et al., 2021; Li et al., 2021). The PCR-based methods are also recommended as confirmation methods for the identification and diagnosis of *V. vulnificus* in the International Organization for Standardization (ISO 21872: 2017) (Hartnell et al., 2019) in addition to the traditional pathogenic culture-based detection methods.

Digital PCR (dPCR), as a representative of the third-generation PCR, is a micro-drop reaction system and assigns templates into 10,000–80,000 independent reaction microchambers, ensuring that the DNA molecules in each microchamber were ≤ 1 (Lei et al., 2021). After PCR amplification, the copy number of the target gene in the initial sample could be calculated directly according to Poisson statistics by collecting the fluorescence signal within each reaction microchamber, which finally achieves the absolute quantification of target DNA without standard curves like qPCR (Wouters et al., 2020). There are two dominant methods of producing microdroplets: the microfluidic chip method represented by the QuantStudio*™* 3D chip digital PCR (cdPCR) system and the “oil-in-water” droplet method represented by the QX100/QX200*™* droplet digital PCR (ddPCR) system. The ddPCR used in this study could partition the reaction mixture into “oil-in-water” microdroplets and disperse them on the reaction area by droplet generator. The reaction mixture and cycling parameters of ddPCR were no different from those of qPCR assay. However, owing to the microtitration of the ddPCR mixture, the shortcoming of qPCR, such as the influence of inhibitory component in mixture (Lei et al., 2020) and the unstable ability to detect weakly positive samples (Suo et al., 2020), had been overcome. A more stable and consistent amplification efficiency could be obtained on ddPCR.

Since the dead bacterial DNA also remained in the samples for a long time (Zeng et al., 2020), routine PCR assays cannot distinguish the target DNA coming from live or dead bacteria in the actual samples (e.g., contaminated food samples after autoclaving and clinical samples after antibiotic treatment), which results in the overestimation of live cells (Zhao et al., 2020). Recently, this bottleneck could be broken by combining photo-reactive DNA-binding dye, such as propidium monoazide (PMA) and ethidium monoazide (EMA), with nucleic acid molecular diagnostics (Codony et al., 2020). A previous study revealed (Copin et al., 2021) that pre-treatment with different concentration gradients of EMA in seafood and environmental samples had a lethal effect on *V. vulnificus* strain compared with PMA. PMA could penetrate into the damaged and dead cells and selectively bind to the DNA in these cells, which can effectively inhibit their PCR amplification (Elizaquível et al., 2014). PMA combined with PCR-based correlation assays had been widely used in the research studies for infectious viable bacterial detection, such as *Brucella* (Zhang et al., 2020), *Escherichia coli* (Deshmukh et al., 2020), *Salmonella* (Xie et al., 2020), and SARS-CoV-2 (Hong et al., 2021).

**FIGURE 1:**
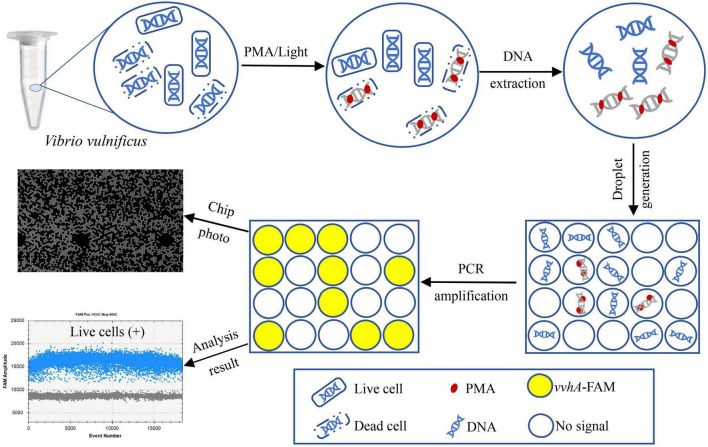
Technical route of PMA-ddPCR for the accurate detection of live cells of *V. vulnificus* by targeting the *vvhA* gene.

To our knowledge, most researchers have developed qPCR assay for detecting *V. vulnificus* (Tsai et al., 2019; Guan et al., 2021; Lee et al., 2021a). However, there were few studies on the detection of *V. vulnificus* by ddPCR, especially based on PMA-ddPCR. In this study, we aimed to establish an accurate and sensitive method based on PMA-ddPCR to detect live cells of *V. vulnificus* in samples (Figure 1). The specificity, sensitivity, and the ability to detect live *V. vulnificus* DNA were analyzed, and the performance in clinical samples was evaluated by testing artificially contaminated plasma sample, compared to that of PMA-qPCR.

## Materials and Methods

### Bacterial Strains and Cultivation

In the PMA-qPCR and PMA-ddPCR assays, optimization and validation assays were performed by using eight reference strains and 19 clinical isolates. Among them, seven strains belonged to *V. vulnificus* (Table 1). Reference strains (*Ref.*) were purchased from the Marine Culture Collections of China (MCCC) and the China General Microbiological Culture Collection Center (CGMCC). Clinical strains (*Clin.*) were isolated from clinical samples in Hwa Mei Hospital, China, and confirmed by an automated microbial identification system. All strains were initially stored in 20% (v/v) glycerol at –80°C. *Vibrio* species strains were grown on 2216E liquid medium (QDRS BIOTEC, China), and the other 13 bacterial strains were cultured on Luria–Bertani (LB) broth medium. All strains were incubated at 37°C for 48 h under constant shaking (180 rpm) to make live cell suspensions for the subsequent experiments. The *V. vulnificus* culturable cells were enumerated by dilution plating on 2216E agar medium at 37°C for 48 h.

**TABLE 1:** Information of bacterial strains used in this study.

Strains	Species	Source	Results	
			PMA- qPCR	PMA- ddPCR
ATCC27562 (*Ref.*)	*Vibrio vulnificus*	MCCC	+ (positive)	+
HM2 (*Clin.*)	*Vibrio vulnificus*	Clinical isolate	+	+
HM3 (*Clin.*)	*Vibrio vulnificus*	Clinical isolate	+	+
HMYJ (*Clin.*)	*Vibrio vulnificus*	Clinical isolate	+	+
HMO2 (*Clin.*)	*Vibrio vulnificus*	Clinical isolate	+	+
HMM16 (*Clin.*)	*Vibrio vulnificus*	Clinical isolate	+	+
HMZ2 (*Clin.*)	*Vibrio vulnificus*	Clinical isolate	+	+
ATCC33653 (*Ref.*)	*Vibrio mimicus*	CGMCC	− (negative)	−
ATCC33812 (*Ref.*)	*Vibrio fluvialis*	MCCC	−	−
ATCC700040 (*Ref.*)	*Vibrio metschnikovii*	CGMCC	−	−
ATCC17749 (*Ref.*)	*Vibrio alginolyticus*	CGMCC	−	−
CGMCC1.997 (*Ref.*)	*Vibrio parahaemolyticus*	CGMCC	−	−
ATCC14100 (*Ref.*)	*Vibrio cholerae*	CGMCC	−	−
CGMCC1.1599 (*Ref.*)	*Vibrio harveyi*	CGMCC	−	−
HMF1 (*Clin.*)	*Klebsiella pneumoniae*	Clinical isolate	−	−
HMF2 (*Clin.*)	*Klebsiella pneumoniae*	Clinical isolate	−	−
HMC13 (*Clin.*)	*Escherichia coli*	Clinical isolate	−	−
HMC14 (*Clin.*)	*Escherichia coli*	Clinical isolate	−	−
HMP96 (*Clin.*)	*Pseudomonas aeruginosa*	Clinical isolate	−	−
HMP97 (*Clin.*)	*Pseudomonas aeruginosa*	Clinical isolate	−	−
HM110 (*Clin.*)	*Staphylococcus aureus*	Clinical isolate	−	−
HM175 (*Clin.*)	*Staphylococcus aureus*	Clinical isolate	−	−
HM7 (*Clin.*)	*Morganella morganii*	Clinical isolate	−	−
HMA57 (*Clin.*)	*Acinetobacter baumannii*	Clinical isolate	−	−
HMA66 (*Clin.*)	*Acinetobacter baumannii*	Clinical isolate	−	−
HM31 (*Clin.*)	*Pseudomonas fluorescens*	Clinical isolate	−	−
HM5 (*Clin.*)	*Enterococcus faecium*	Clinical isolate	−	−

*ATCC, American Type Culture Collection; HM, Hwa Mei Hospital; CGMCC, China General Microbiological Culture Collection Center; Ref., reference strains; Clin., clinical strains isolated from clinical samples in Hwa Mei Hospital, China.*

### Preparation of Heat-Killed Cell Suspensions

We divided the counted bacterial solutions in Section “Bacterial Strains and Cultivation” into two equal portions: One was used to prepare live *V. vulnificus* cell suspensions and the other was incubated at 95°C for 10 min to obtain the heat-killed cell suspensions. The effect of heat killing was assessed by spreading 100 μL heat-killed cell suspension on 2216E agar medium and repeating for three plates. No colony formation after incubation at 37°C for 48 h proved that all *V. vulnificus* cells were dead.

### Genomic DNA Extraction

The genomic DNA was extracted with a MiniBEST Bacterial Genomic DNA Extraction Kit (TaKaRa, Japan) according to the user manual. DNA was eluted in 80 μL nuclease-free water. The concentration and quality of extracted DNA were analyzed using a Nanodrop ND-1000 spectrophotometer (Thermo Scientific, United States). All template DNA were stored at −40°C until use.

### Primer and Probe Design

Multiple sequence alignments were performed on the *vvhA* gene sequence based on the published sequences from GenBank (Accession nos.: KC821520.1, MW132717.1, AF376032.1, KU680790.1, KP224256.1, KF255393.1, and CP0112740.1), and the similarity regions among those sequences were entered into the National Center for Biotechnology Information (NCBI) Primer-BLAST (Basic Local Alignment Search Tool) for primer design. At the same time, for probe design, the internal hybridization oligo-parameters in Primer-BLAST was chosen and performed with the following criteria: (1) The probe size was set as minimum at 18–30 bp; (2) the melting temperature (*Tm*) was set between 50 and 70°C; (3) and the GC content was set as 40–60%. Other parameters were set as default. The probe had a 6-FAM (6-carboxy-fluorescein) at the 5′ end and a BHQ1 (black hole quencher 1) at the 3′ end. All primers and probe used in this study are listed in Table 2 and synthesized at Sangon Biotech, China.

**TABLE 2:** Primer and probe sequences in this study.

Primer/Probe	Sequence (5′-3′)	Product size
F1	TGGCACGGGTATTCATTTGG	103 bp
R1	TAACTGCTGGCGAATGGACCAATG	
F2	CTGACGCCAAAATTGTCCGTT	126 bp
R2	CAATGTAAGTGCGGCGGTTT	
F3	CCCGTTTCTGGTTACACAC	126 bp
R3	TTCACTTCCGCACCTACTT	
F4	TTGTCCGTTTCACCGTCGAT	144 bp
R4	AACGGGTTTCACCCAAAGGT	
F5	AATTGTCCGTTTCACCGTCG	115 bp
R5	AATGTAAGTGCGGCGGTTTG	
F6	TGACGCCAAAATTGTCCGTT	148 bp
R6	GTCGTAACTGCTGGCGAATG	
P6	6-FAM-TGCCGACAAGCCTGGCACGGGT-BHQ1	

*F, forward primer; R, reverse primer; P, probe; 6-FAM, 6-carboxy-fluorescein; BHQ1, black hole quencher 1.*

### Optimization of the Primer Pairs and Probe

The performance of primers and probes is key to evaluating the quality of nucleic acid amplification. Not all primer pairs screened by Primer-BLAST could produce stable positive signals. We used qPCR assay to screen the optimal primer pairs and designed the corresponding probe for subsequent PMA-qPCR and PMA-ddPCR assays.

### Quantitative Real-Time PCR

The reaction was carried out in a total volume of 20 μL, containing 10 μL of a PerfectStart II Probe qPCR SuperMix (Trans, China), 1 μL of primer (10 μM), 1 μL of probe (10 μM), 2 μL of template DNA, and 5 μL of nuclease-free water. The cycling parameters were performed as follows: 95°C for 30 s (pre-denaturation), followed by 40 cycles of 95°C for 5 s (denature), 65°C for 30 s (annealing and extension), and then cooling to 50°C for 30 s. The qPCR assay was performed on the LightCycler 480 thermocycler (Roche, United States).

To increase the detection accuracy and effectiveness, the concentration of primer and probe and the annealing temperature were optimized. Totally, five concentrations of the primer (0.1, 0.2, 0.3, 0.4, and 0.5 μM) were tested together with four concentrations of the probe (0.3, 0.4, 0.5, and 0.6 μM), and then five different annealing temperatures (55, 57, 60, 63, and 65°C) were tested. The optimal qPCR protocol was judged by the threshold cycle (*Ct*) and the fluorescence intensity enhancement (Δ*Rn*) of the amplification curves under different amplification conditions. The qPCR parameter optimization was performed with two independent replicates.

### Droplet Digital PCR

The ddPCR was carried out in a total volume of 14 μL, containing 7 μL of the PerfectStart II Probe qPCR SuperMix (Trans, China), 1.4 μL of each primer (10 μM), 0.42 μL of probe (10 μM), 1.4 μL of template DNA, and 2.38 μL of nuclease-free water. The reaction mixtures were injected into the spiking well on chip. Pipettes were used to drain the air bubbles in spiking wells, and then the spiking wells were covered with silica caps. Subsequently, the chip was put into the ddPCR instrument. Approximate 30000 droplets were produced and assigned on a digital chip using a droplet generator (Pilot Gene, China). Then, an independent PCR was conducted to end point using the Thermo Cycler PCR (Pilot Gene, China) on chip, and the cycling parameters were performed as qPCR in 2.6. Following PCR amplification, the chip was transferred to a chip scanner (Pilot Gene, China) for the reading and analysis of fluorescence amplitudes in each droplet.

To increase the ddPCR detection accuracy and effectiveness, the concentration of primers/probe and the annealing temperature were optimized. Totally, three concentrations of the primer (0.6, 0.8, and 1.0 μM) were tested together with three concentrations of the probe (0.2, 0.3, and 0.4 μM), and then three annealing temperatures (58, 60, and 62°C) were tested. The optimal ddPCR protocol was judged by the degree of separation between all positive and negative spots in the amplification images and the amplitude of the positive cluster. The ddPCR parameter optimization was performed using three independent replicates.

### PMA Treatments

PMA (Biotium, United States) was dissolved in 20% dimethyl sulfoxide (Solarbio, China) to generate a stock concentration of 10 mM and stored at −40°C in the dark. Optimization of the PMA treatment was conducted at the room temperature of 25°C. To find the minimum PMA concentration that can completely inhibit the amplification of DNA from dead cells without affecting that from live cells of *V. vulnificus*, a series of concentrations (0, 10, 20, 40, 60, 100, 140, and 180 μM) of PMA were added to live cell suspensions and heat-killed cell suspensions, following an incubation period of 15 min in the dark. Then, the samples were exposed for 20 min using a 200 W blue LED lighting array with an output wavelength of 465 nm. Under the optimal PMA concentration, different exposure times (0, 1, 3, 5, 10, 15, 20, 25, and 30 min) were tested on heat-killed cell suspensions to find the optimal exposure time. PMA parameter optimization was performed with three independent replicates.

### Clinical Sample Detection

To validate the diagnostic performance of PMA-ddPCR in clinical samples compared to that of PMA-qPCR, a series of plasma samples artificially spiked with different concentrations of *V. vulnificus* were used to simulate the clinical samples infected by *V. vulnificus*. The spiked samples were treated with PMA and extracted genomic DNA with a MiniBEST Bacterial Genomic DNA Extraction Kit (TaKaRa, Japan). Clinical sample detection assays were performed using three independent replicates.

### Statistical Analysis

The LOD is the target level that would be detected in 95% of cases (Zhu et al., 2021). In this study, probit regression analysis was used to fit the limit of detection (LOD) of PMA-qPCR and PMA-ddPCR. The positive rates obtained by testing the *V. vulnificus* samples of different concentrations were transformed into probability values using probability function. The analysis of variance (ANOVA) was applied to test whether the effect of the primer/probe concentration (section “Optimization of qPCR Amplification Conditions”) or the PMA concentration (section “Screening the Optimal Concentration and Light-Exposed Time of PMA Treatment”) based on the *Ct* value was significant or not. The results of the positive and negative samples from the corresponding assays were used to perform receiver operating characteristic (ROC) curve analysis. ROC curve analysis was used to calculate the maximum value of the Youden index and cut-off value. The cut-off value and the calibration curve in sensitivity analysis were used to determine the limit of quantitation (LOQ) of PMA-qPCR and PMA-ddPCR (Nutz et al., 2011). The probit regression analysis and ANOVA were performed using IBM SPSS Statistics 23 software. ROC curve analysis and calibration curve in sensitivity analysis were performed by GraphPad Prism 8.0 software. All figures were drawn by using GraphPad Prism 8.0 software.

## Results

### Screening of Specific Primers and Probes

Totally, six pairs of potential primers were designed by Primer-BLAST and are listed in Table 2. Comparing the *Ct* value and Δ*Rn* in amplification curves (Supplementary Table 1), the primer pair (F6/R6) showed the best performance. Therefore, F6/R6 and the corresponding probe (P6) were selected for subsequent qPCR and ddPCR assays.

### Optimization of qPCR Amplification Conditions

To optimize the conditions of qPCR amplification, a total of 20 primer/probe concentration combinations in Supplementary Table 2 were compared. The *Ct* values obtained from these combinations were subjected to two-way ANOVA. The results indicated that there was no statistically significant interaction for “Primer*Probe” (*P* > 0.05). There was also no statistically significant difference among probe concentrations (*P* > 0.05), but there were statistically significant differences among primer concentrations (*P* < 0.05). For further screening the optimal primer concentration, the Tukey’s *post hoc* test was performed, and the estimated marginal mean of *Ct* value was calculated. The results showed that “Primer = 0.5 μM” was the optimal group. In this group, there was the highest Δ*Rn* when the concentration of probe was 0.5 μM. So, the combination (primer = 0.5 μM and probe = 0.5 μM) was selected as the optimal primer/probe concentration combination (Supplementary Table 2). Thereafter, five different annealing temperatures were tested (Supplementary Table 3). In the subsequent qPCR assays, 65°C, at which the highest detection accuracy can be achieved, was chosen as the optimal annealing temperature.

### Optimization of ddPCR Amplification Conditions

**FIGURE 2:**
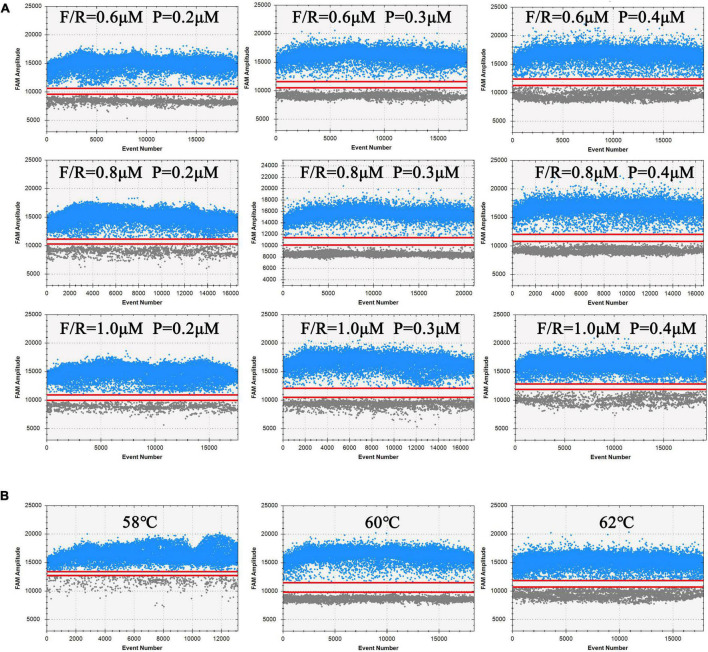
Analysis results of ddPCR under different processing conditions. Blue dots represent positive droplets, and gray dots represent negative droplets. The figures show the most available results of three independent replicates. **(A)** Optimization of primers and probe concentration combinations on ddPCR. **(B)** Optimization of annealing temperature on ddPCR.

The amplification efficiencies of different primers and probe concentration combinations were compared in ddPCR assay. The results in Figure 2A reveal that the clusters in the combination (F/R = 1 μM, *P* = 0.3 μM) were divided clearly (largest separation of the two red lines) with highest fluorescence amplitudes. Hence, this combination was chosen as the optimal primer/probe concentration combination. Similarly, as shown in Figure 2B, the annealing temperature of 60°C was considered to be the optimum, with the largest discrimination in fluorescence intensity between blue and gray dots.

### Screening the Optimal Concentration and Light-Exposed Time of PMA Treatment

**FIGURE 3:**
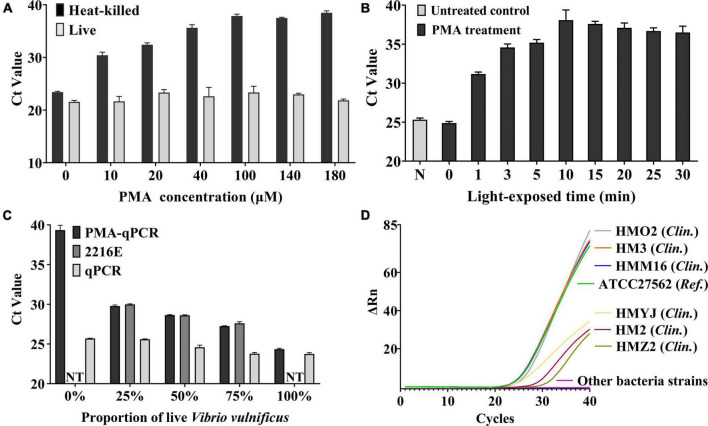
**(A)** Optimization of final PMA concentration. The different concentrations (10–180 μM) of PMA were treated on live *V. vulnificus* cell suspensions and heat-killed cell suspensions. Error bars represent the standard deviation based on three replicates. **(B)** Optimization of light-exposed time. The different light-exposed times were treated on heat-killed cell suspensions. N: PMA untreated control. Error bars represent the standard deviation based on three replicates. **(C)** Verification of the ability to detect live *V. vulnificus* cells by PMA-qPCR. The different concentrations (0–100%) of live cells were mixed with heat-killed cells and treated with PMA (PMA-qPCR group) or not (qPCR group). While the 2216E group was the blank control which was the corresponding concentration of live cells mixed with 2216E liquid medium. NT, no test. When the proportion of live *V. vulnificus* was 0% and 100%, the samples were pure dead bacterial solution or live bacterial solution, and it is not necessary to mix with 2216E medium. Error bars represent the standard deviation based on three replicates. **(D)** Specificity test of PMA-qPCR. Only the above seven *V. vulnificus* strains were observed positive signals. *Clin.*, clinical strain of *V. vulnificus* isolated from clinical samples; *Ref.*, reference strain of *V. vulnificus*.

The different concentrations and light-exposed time of PMA treatment had different effects on the cells (Wu et al., 2015). Considering the unknown initial concentration of bacteria in actual samples, we chose a concentration (2.65 × 10^8^ CFU/mL) for the following experiments. As shown in Figure 3A, for the heat-killed cell group, as the concentration increased, the *Ct* value gradually raised and leveled off at 100 μM. For live cell group, there was no statistical significance (one-way ANOVA, *P* > 0.05) in the *Ct* value under different concentrations of PMA treatment, which showed that the different concentrations (0–180 μM) of PMA treatment had no inhibitory effect on amplification of DNA from live cells of *V. vulnificus*. The results comparing the different light-exposed times during PMA treatments are shown in Figure 3B. The *Ct* value is highest at 10 min, which improved that this light-exposed time had the strongest inhibitory effect on the dead *V. vulnificus* DNA amplification. These results indicated that the optimal strategy for this study was to treat the samples with PMA at a final concentration of 100 μM and expose them for 10 min afterward, which could completely inhibit the DNA amplification from the dead cells of *V. vulnificus*.

### Verification of the Ability to Detect Live Cells of *Vibrio vulnificus*

We used the qPCR assay to assess the ability to detect live cells of *V. vulnificus* under the optimal condition of PMA treatment. The different concentrations of live cells were mixed with heat-killed cells and treated with PMA (PMA-qPCR group) or not (qPCR group). The corresponding concentration of live cells mixed with 2216E liquid medium was used as the blank control (2216E group). As shown in Figure 3C, the qPCR group showed the results of DNA amplification from total cells (dead and live cells). PMA-qPCR and 2216E groups reflected the status of DNA amplification only from live cells. As the live cells of *V. vulnificus* cells increased in samples, the *Ct* values of the two groups decreased accordingly, and were close to each other. The aforementioned results indicated that the condition of PMA treatment used in this study was capable of detecting live cells of *V. vulnificus*.

### Specificity of PMA-qPCR and PMA-ddPCR

To evaluate the specificity of PMA-qPCR and PMA-ddPCR, the genomic DNA isolated from *V. vulnificus* and non-*V. vulnificus* strains (Table 1) were tested, respectively. The positive signals were only observed in the *V. vulnificus* reference strain and six clinical strains, while other strains (non-*V. vulnificus*) showed negative signals (Figure 3D).

### Sensitivity and Regression Analysis of PMA-qPCR and PMA-ddPCR

**FIGURE 4:**
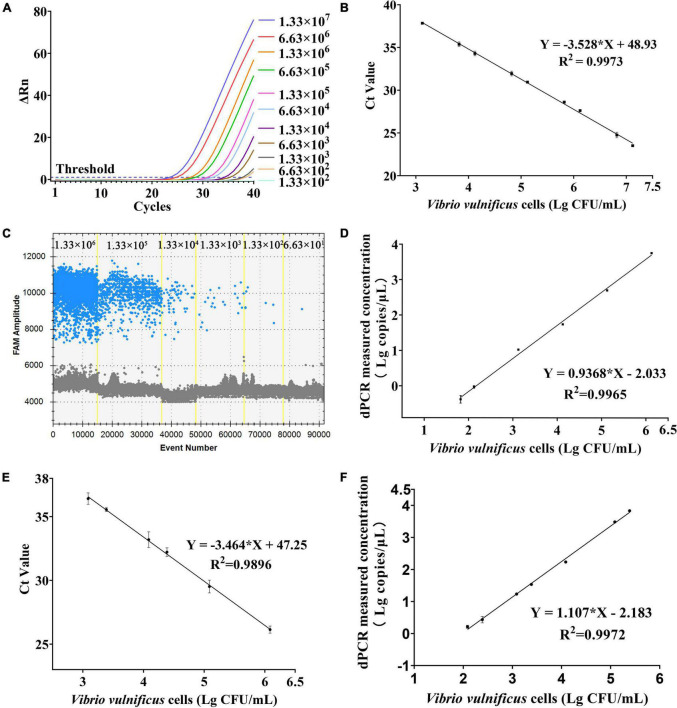
Sensitivity and regression analysis of PMA-qPCR and PMA-ddPCR. **(A)** Amplification curves of different concentrations of *V. vulnificus* from pure culture on qPCR. **(B)** Linear regression analysis based on qPCR in pure culture solutions. Error bars represent the standard deviation based on three replicates. **(C)** Picture of FAM channel from different concentrations of *V. vulnificus* in pure culture on ddPCR. **(D)** Linear regression analysis based on ddPCR in pure culture solutions. Error bars represent the standard deviation based on three replicates. **(E)** Linear regression analysis based on qPCR in plasma samples. Error bars represent the standard deviation based on three replicates. **(F)** Linear regression analysis based on ddPCR in plasma samples. Error bars represent the standard deviation based on three replicates.

For sensitivity, a series of dilution of *V. vulnificus* solutions approximately ranging from 1.33 × 10^1^ to 1.33 × 10^7^ CFU/mL were, respectively, detected by PMA-qPCR and PMA-ddPCR (Table 3). The amplification curves of qPCR and ddPCR amplification images with different concentrations of *V. vulnificus* are shown in Figures 4A,C, and the constructed standard curve is shown in Figures 4B,D. The LOD was determined by the fitting of a probit regression analysis. The results showed the LODs of PMA-qPCR and PMA-ddPCR in detecting *V. vulnificus* from the pure culture were, respectively, 1.14 × 10^3^ and 29.33 CFU/mL (Supplementary Figure 1). The LOQ was defined as the amount of DNA corresponding to the *Ct* value or the ddPCR measurement value (copies/μL) at which the sum of specificity and sensitivity of the assay was maximized (optimal cut-off point in ROC curve) and calculated using the regression equation, the Youden index, and the cut-off value. The results showed the LOQ was 1.29 × 10^3^ CFU/mL (PMA-qPCR) and 53.64 CFU/mL (PMA-ddPCR) in pure culture bacterial solutions.

**TABLE 3:** Comparison of detection results of PMA-qPCR and PMA-ddPCR in pure culture solutions.

Plate count (CFU/mL)	PMA-qPCR (Ct)	PMA-ddPCR (Copies/μL)
1.33 × 10^7^	23.51 ± 0.08	−
1.33 × 10^6^	27.62 ± 0.04	5699.22 ± 58.13
1.33 × 10^5^	30.94 ± 0.12	503.01 ± 3.53
1.33 × 10^4^	34.30 ± 0.22	55.17 ± 0.04
1.33 × 10^3^	37.84 ± 0.08	10.60 ± 0.46
1.33 × 10^2^	−	0.94 ± 0.06
6.63 × 10^1^	−	0.43 ± 0.08
1.33 × 10^0^	−	−

*“−”: No valid data.*

### Performance of PMA-qPCR and PMA-dPCR to Detect *Vibrio vulnificus* in Spiked Plasma Samples

The performance of PMA-ddPCR to detect *V. vulnificus* in spiked plasma samples was compared to that of PMA-qPCR (Table 4). The results are shown in Figure 4E (PMA-qPCR) and Figure 4F (PMA-ddPCR), and the LODs were 1.00 × 10^3^ CFU/mL (PMA-qPCR) and 65.20 CFU/mL (PMA-ddPCR) (Supplementary Figure 2). The results were consistent with those of pure culture bacterial solution, and it confirmed that PMA-ddPCR was applicable for the determination of *V. vulnificus* in clinical samples.

**TABLE 4:** Comparison of detection results of PMA-qPCR and PMA-ddPCR in spiked plasma samples.

Plate count (CFU/mL)	PMA-qPCR (Ct)	PMA-ddPCR (Copies/μL)
1.22 × 10^6^	26.14 ± 0.23	−
2.44 × 10^5^	NT	6784.44 ± 80.90
1.22 × 10^5^	29.52 ± 0.41	3043.05 ± 88.28
1.22 × 10^4^	33.19 ± 0.50	170.83 ± 0.91
2.44 × 10^3^	35.55 ± 0.12	34.19 ± 0.61
1.22 × 10^3^	36.41 ± 0.37	17.07 ± 0.38
2.44 × 10^2^	−	2.76 ± 0.51
1.22 × 10^2^	−	1.63 ± 0.15
1.22 × 10^1^	−	−

*“−”: No valid data. NT, not test.*

## Discussion

Using the traditional pathogenic culture to detect *V. vulnificus* required pre-enrichment, isolation and purification, biochemical identification, and serological identification, which would take 3–7 days. The traditional culture method is prone to give false-negative results, especially in distinguishing *Vibrio* homologous strains and VBNC cells. Meanwhile, the atypical clinical signs of *V. vulnificus* infection with increasingly unclear factors of exposure risk have caused difficulties in its early diagnosis and treatment (Liang et al., 2021). With the development of molecular diagnosis technology, qPCR based on nucleic acid fragment detection has provided a rapid, efficient, and specific way to detect *V. vulnificus* (Abioye et al., 2021; Lee et al., 2021a; Álvarez-Contreras et al., 2021; Bonny et al., 2022). However, there is no official authority that can provide *V. vulnificus* DNA calibrations with clear quantitative values. Moreover, some factors, such as the quality of primers and probes, the inhibitory components in the samples, the laboratory environment, and the experimental skills of the operator, could directly affect the quality of the standard curve in qPCR assay, which limit the accuracy of qPCR in the practical detection. Digital PCR, as a representative of the third-generation PCR assay, does not need to rely on standard curves and *Ct* values for quantification, which could improve the accuracy of detection compared with qPCR (Lei et al., 2021). In this study, we developed a ddPCR method to detect *V. vulnificus* and evaluated the performance of this method in plasma samples, which would provide valuable insights for accurate detection of *V. vulnificus* in future.

Pathogens only pose a human health risk if viable (Wang et al., 2021). The genomic DNA of the pathogen remains in the body for a long time after the patient has been treated with antibiotics (Kobayashi et al., 2010), which made it difficult to effectively distinguish the DNAs from dead cells and those from live cells by traditional PCR assay. The DNAs from dead cells could lead to an overestimation of live cell counts and produce a false-positive result, which is not conducive to clinical monitoring of *V. vulnificus* infection. Since the first study on using PMA to differentiate live cells from dead cells was reported in 2006 (Nocker et al., 2006), PMA has become one of the most widely used dyes (Codony et al., 2020) and been applied to many fields of microbiology (Deshmukh et al., 2020; Cechova et al., 2021; Hong et al., 2021). PMA could not bind to the DNA of live cells with the intact bacterial plasma membrane structure. Therefore, the PMA treatment with PCR (PMA-PCR) is considered a successful approach to detect live cells (Lazou et al., 2021), reducing the rate of misdiagnosis in the detection of pathogen infection.

The suitable conditions of PMA treatment are key to conduct PMA-PCR as overtreatment could damage live cells and lead to wrong results (Young et al., 2017). The concentration of PMA and light-exposed time during PMA treatment are two important factors that should be optimized according to the target strain, initial concentration, exposure lamps, etc. In this study, the optimal strategy to distinguish the live cells from the dead cells of *V. vulnificus* was to treat the samples with 100 μM PMA for 15 min in the dark and expose them to 465 nm blue LED lighting array for 10 min. We mixed live cells with dead cells of *V. vulnificus* in different proportions (0–100%) and detected the *Ct* value by qPCR. The results verified the ability to detect live cells of *V. vulnificus* by PMA-qPCR and also revealed that the PMA concentration (100 μM) we chose had no effect on live cells. So, we believed that the combination of PMA treatment and detection of DNA by PCR (qPCR or ddPCR) would provide a reference for the evaluation of the therapeutic effect on clinical *V. vulnificus* infections.

It is necessary to choose proper target gene for successful detection of *V. vulnificus* in samples. An exocrine cytotoxin hemolysin A encoded by the single-copy *V. vulnificus* hemolysin A gene (*vvhA*) is an essential virulence factor causing cell infection and tissue damage by forming small pores on the cell membrane surface of target cells (Guan et al., 2021). Therefore, the *vvhA* gene has been considered the important virulence marker of *V. vulnificus* (Yuan et al., 2020). The *vvhA* gene is present in the majority of *V. vulnificus* strains that are closely related to human diseases (Wong et al., 2005; Baker-Austin and Oliver, 2020; Lin et al., 2021) and is often used as a marker for the detection of *V. vulnificus* (Wei et al., 2014; Gyraite et al., 2019; Bullington et al., 2022). Thus, in this study, we chose the *vvhA* gene as the target gene and tested its performance of PMA-qPCR and PMA-ddPCR on pure cultures and plasma samples. The results showed only the positive signals were observed in those strains of *V. vulnificus*, and there was no cross-reactions with DNA from no-*V. vulnificus* strains. This clearly validated that the *vvhA* gene is suitable for *V. vulnificus* detection. As well known, the standard for *V. vulnificus* detection is a culture-based method. So, based on the results of our experiments (Supplementary Table 4), we evaluated the agreement between PMA-ddPCR developed in this study and traditional culture-based method by using the kappa test. The results from the kappa test showed that there is a good agreement between the results of the PMA-ddPCR method and culture-based method (kappa = 0.800). Considering the sensitivity, the LOD of the PMA-ddPCR in this study was 29.33 CFU/mL, which performed better than those methods in previous studies, such as the LOD of *V. vulnificus*, was 1 × 10^3^ CFU/mL with qPCR (Panicker and Bej, 2005), or 1.2 × 10^2^–1 × 10^3^ CFU/mL with recombinase polymerase amplification (RPA) (Yang et al., 2020; Zhu et al., 2021), or 2 × 10^3^ CFU/mL with loop-mediated isothermal amplification (LAMP) (Zhang et al., 2018). In accurate detection in artificially spiked plasma samples, the performance of the PMA-ddPCR was also superior to that of PMA-qPCR (Supplementary Figure 2), which means that PMA-ddPCR is more capable in handling clinical samples. What Regrettably, the samples with higher concentrations of *V. vulnificus* cells (greater than 10^6^ CFU/mL) needed to be diluted before detection because of the limitation of “oil-in-water” microdroplet number on PMA-ddPCR. Otherwise, the microdroplets would be oversaturated (target DNA molecules in each microchamber ≥ 1), leading to no valid results (Table 3). So, the microdroplet number led to a lower upper limit of the detection for ddPCR than that of qPCR. In brief, in our study, the PMA-ddPCR is suitable for the detection of *V. vulnificus* at the range from 1.33 × 10^6^ to 6.63 × 10^1^ CFU/mL in samples. It is more sensitive and accurate in the detection of target DNA at low copy numbers.

It is worth noting that the measured concentration of *V. vulnificus* by PMA-ddPCR was 4–7 times higher than that using by the conventional plate counting method in the theoretical situation in this study (Table 3). Which method is more accurate? It may be PMA-ddPCR. There may be two reasons as follows for this question. First, as a technology of absolute quantification, ddPCR did not rely on the calibration curve. Meanwhile, the divided droplets in ddPCR could minimize the interference of inhibitory components during the amplification of target DNA, enhancing the accuracy of ddPCR results to a large extent. Second, some inherent disadvantages of the agar plate culture, such as the overlapping of multiple colonies during growth, the inability to cultivate *V. vulnificus* in the VBNC state, or the decline of bacterial growth activity often led to the lower counts of *V. vulnificus* by using the conventional plate counting method.

## Conclusion

In summary, an assay combining ddPCR with PMA (PMA-ddPCR) to accurately detect the live cells of *V. vulnificus* based on *vvhA* gene was successfully established and evaluated in artificially contaminated plasma samples. The PMA-ddPCR assay developed in our study provides a reliable tool for the clinical diagnosis and the evaluation of the therapeutic effect in *V. vulnificus* infection, which is very important in *Vibrio* illness surveillance.

## Data Availability Statement

The raw data supporting the conclusions of this article will be made available by the authors, without undue reservation.

## Author Contributions

LH: methodology, data curation, software, and writing—original draft preparation. YF: data curation and software. SZ: investigation. ZP: investigation and data curation. JX: software. PZ: conceptualization writing – reviewing and editing and supervision. JG: supervision. All authors contributed to the final version of the manuscript and approved the final manuscript.

## Conflict of Interest

JX is employed by Pilot Gene Technologies (Hangzhou) Co., Ltd. The remaining authors declare that the research was conducted in the absence of any commercial or financial relationships that could be construed as a potential conflict of interest.

## Publisher’s Note

All claims expressed in this article are solely those of the authors and do not necessarily represent those of their affiliated organizations, or those of the publisher, the editors and the reviewers. Any product that may be evaluated in this article, or claim that may be made by its manufacturer, is not guaranteed or endorsed by the publisher.
