# Supplementary material for: An Assay Combining Droplet Digital PCR With Propidium Monoazide Treatment for the Accurate Detection of Live Cells of Vibrio vulnificus in Plasma Samples
Source: Front Microbiol. 2022 Jul 15;13:927285. doi: 10.3389/fmicb.2022.927285 (PMC9335127; doi:10.3389/fmicb.2022.927285)
Supplement: Supplementary file 1 [file Data_Sheet_1.docx]

Supplementary Material

**Supplementary Table 1.** The Ct and *ΔRn* of different primer pairs.

|  | F1/R1 | F2/R2 | F3/R3 | F4/R4 | F5/R5 | F6/R6 |
| --- | --- | --- | --- | --- | --- | --- |
| Ct | 15.45 | 15.91 | 15.13 | 16.42 | 15.32 | 15.39 |
| *ΔRn* | 61.63 | 77.51 | 62.87 | 73.57 | 53.79 | 82.20 |

**Supplementary Table 2.** The *Ct* value and mean *ΔRn* with different primer and probe concentration.

| Primer (μM) | | Probe (μM) | | | |
| --- | --- | --- | --- | --- | --- |
|  |  | 0.3 | 0.4 | 0.5 | 0.6 |
| 0.1 | *Ct_1_/ Ct_2_* | 21.59/21.22 | 20.85/20.77 | 21.74/20.5 | 21.9/20.97 |
|  | *ΔRn* | 37.19 | 37.49 | 31.37 | 33.68 |
| 0.2 | *Ct_1_/ Ct_2_* | 19.3/20.13 | 20.9/20.42 | 20.89/20.79 | 20.94/20.54 |
|  | *ΔRn* | 52.47 | 57.81 | 62.53 | 64.97 |
| 0.3 | *Ct_1_/ Ct_2_* | 21.78/21.11 | 20.73/20.68 | 20.58/20.58 | 20.66/21.74 |
|  | *ΔRn* | 60.24 | 76.89 | 81.56 | 71.88 |
| 0.4 | *Ct_1_/ Ct_2_* | 21.53/20.69 | 21/20.9 | 20.8/20.87 | 20.32/21.58 |
|  | *ΔRn* | 69.25 | 78.20 | 85.96 | 78.31 |
| 0.5 | *Ct_1_/ Ct_2_* | 20.94/20.99 | 20.58/20.33 | 19.8/20.35 | 20.62/20.1 |
|  | *ΔRn* | 76.95 | 90.81 | 101.10 | 95.50 |

**Supplementary Table 3.** The *Ct* and *ΔRn* of annealing temperature between 55-65 ℃.

|  | 55 ℃ | 57 ℃ | 60 ℃ | 63 ℃ | 65 ℃ |
| --- | --- | --- | --- | --- | --- |
| *Ct* | 21.03 | 20.71 | 19.95 | 19.93 | 19.68 |
| *ΔRn* | 89.91 | 95.57 | 93.81 | 97.08 | 99.42 |

**
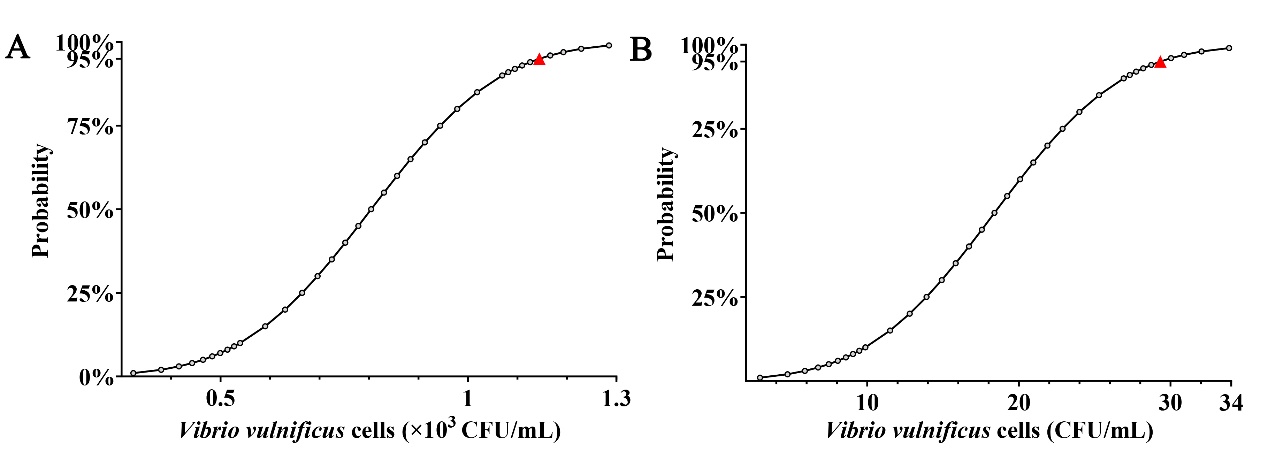
 Supplementary Figure 1.** Probit regression analysis of PMA-qPCR and PMA-ddPCR in detecting *V. vulnificus* from the pure culture, Triangle, LOD. **(A)** The data collected from PMA-qPCR, **(B)** The data collected from PMA-ddPCR.


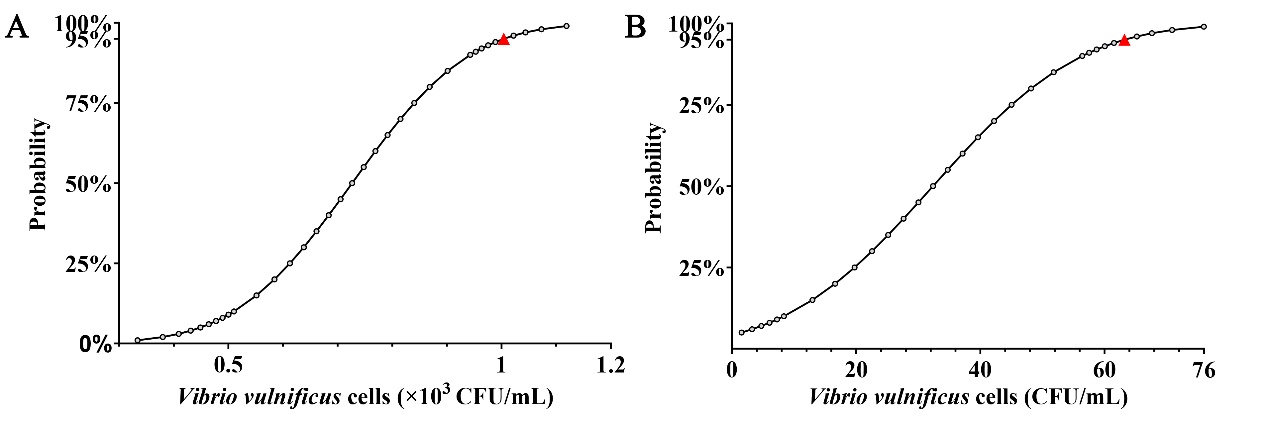


**Supplementary Figure 2.** Probit regression analysis of PMA-qPCR and PMA-ddPCR in detecting *V. vulnificus* from spiked plasma samples, Triangle, LOD. **(A)** The data collected from PMA-qPCR. **(B)** The data collected from PMA-ddPCR.

**Supplementary Table 4. The results of PMA-ddPCR method and culture-based method.**

| Culture-based method | PMA-ddPCR | |
| --- | --- | --- |
|  | Negative | Positive |
| Negative | 20 | 0 |
| Positive | 6 | 39 |

Kappa = 0.800, approximate significance < 0.001, which Showed that there is a good agreement between the results of PMA-ddPCR method and culture-based method.
